# Factor V Leiden G1691A and prothrombin G20210A mutations among Palestinian patients with sickle cell disease

**DOI:** 10.1186/s12878-018-0097-0

**Published:** 2018-01-16

**Authors:** Fekri Samarah, Mahmoud A. Srour

**Affiliations:** 1 Department of Medical Technology, Faculty of Allied Health Sciences, Arab American University in Jenin, Jenin, Palestine; 2 Department of Medical Laboratory Sciences, Faculty of Health professions, Al-Quds University, Jerusalem, Palestine; 3 Present address: Department of Biology & Biochemistry, Faculty of Science, Birzeit University, Birzeit, Palestine

**Keywords:** Factor V Leiden, Prothrombin G20210A, Sickle cell disease, Palestine

## Abstract

**Background:**

Vascular thrombosis is an important pathophysiological aspect of sickle cell disease (SCD). This study aimed to investigate the prevalence and clinical impact of factor V Leiden G1691A (FVL) and prothrombin G20210A mutations among Palestinian sickle cell disease (SCD) patients.

**Methods:**

A total of 117 SCD patients, including 59 patients with sickle cell anemia (SS), 33 patients with sickle β-thalassemia and 25 individuals with sickle cell trait (AS) were studied. The control group consisted of 118 healthy individuals. FVL and prothrombin G20210A mutations were determined by RFLP PCR.

**Results:**

Analysis of the clinical history of SCD patients revealed that seven patients have had vascular complications such as ischemic stroke or deep vein thrombosis. In SCD patients, the inheritance of the FVL mutation showed a significantly higher incidence of pain in joints, chest and abdomen as well as regular dependence on blood transfusion compared to SCD with the wild type. Age- and sex-adjusted logistic regression analysis revealed a significant association between FVL and sickle cell anemia with an odds ratio (OR) of 5.6 (95% confidence intervals [CI] of 1.91–39.4, *P* = 0.039) in SS patients. However, increased prevalence of the FVL in AS subjects and sickle β-thalassemia patients was not statistically significant compared to controls (OR 3.97, 95% CI 0.51–28.6, *P* = 0.17 and OR 3.59, 95% CI 0.35–41.6, *P* = 0.26, respectively). The distribution of prothrombin G20210A mutation among SCD patients compared to controls was not significantly different, thus our findings do not support an association of this mutation with SCD.

**Conclusions:**

FVL was more prevalent among SS patients compared to controls and it was associated with higher incidence of disease complications among SCD patients.

## Background

Sickle cell disease (SCD) is an inherited disorder of β-globin gene and HbS was one of the first structural variants of hemoglobin to be discovered. HbS results from the substitution of valine for glutamic acid at the sixth codon of β-globin gene. SCD is a term used to describe not only homozygous HbSS but also includes cases of compound heterozygotes with other β-globin gene disorders such as β-thalassemia (β^0^ or β^+^) [1]. Although sickle hemoglobin is the product of one mutated gene, the disease phenotype is multigenetic, and gene polymorphisms dictate the different variations seen in SCD patients [2]. The contribution of inherited thrombophilia to the pathophysiology of SCD has been tackled by many reports. The mortality rate of SCD patients has been shown to increase with the presence of acute chest syndromes, as well as occlusive strokes [3]. In addition, a hypercoagulable state in SCD patients has been established that is attributed to several factors including the interaction of sickle cells with the endothelium. The adherence of sickle cells to endothelium is attributed to the increased levels of circulating interleukins as well as platelet and monocyte activation [4].

The coinheritance of genetic thrombophilia thus may exacerbate the hypercoagulable state in SCD patients. The identification of SCD patients with risk factors for developing thrombosis should be valuable in the management and prevention of vasoocclusive crisis through pharmacological intervention using antithrombotic medications [5].

A well-established genetic predisposition to venous thrombosis that occurs in approximately 5% of Caucasian populations is a single-point mutation in the gene encoding coagulation factor V (G1691A) or Factor V Leiden (FVL). FVL is associated with a 7-fold increased risk of venous thrombosis in heterozygote individuals and a 50-fold to 100-fold increased risk of venous thrombosis in homozygote individuals [6]. The second most common risk factor for venous thrombosis is the prothrombin G20210A mutation which is also specific for Caucasian populations (2%) [7]. The prothrombin G20210A mutation is associated with a higher plasma prothrombin levels and a three-fold greater risk of venous thrombosis.

In this study, the prevalence of FVL G1691A and prothrombin G20210A mutation was assessed in 117 SCD individuals and 118 healthy controls from the West Bank of Palestine.

## Methods

### Study design and subjects

A cross-sectional study was conducted with the objective to investigate the prevalence and clinical impact of factor V Leiden G1691A (FVL) and prothrombin G20210A mutations among Palestinian (of Caucasian race) sickle cell disease (SCD) patients. Sickle cell and sickle β-thalassemia patients were all recruited from Al-Watani Hospital in Nablus. This Hospital is the referral center for these disorders in Northern Governorate of Palestine. And the majority of Sickle cell anemia cases in Palestine are registered in the Northern Governorates of Palestine. For selection of the patients, the medical files of patients registered as Sickle cell anemia or Sickle β-thalassemia (S/βthal) at Al-Watani Hospital were reviewed and patients that fulfilled the inclusion criteria were contacted and asked to participate. Information about the health status or clinical complications was collected from medical files. In addition, all patients who accepted to participate in the study were asked to to state their age, sex and confirm their diagnosis using an interview-based questionnaire. The inclusion criteria were: confirmed diagnosis of Sickle cell anemia, Sickle cell trait, or S/βthal, did not experience vascular crisis or chest syndrome at time of sampling, did not show thrombotic events or have family history of thrombosis, and were not transfused during the last 4 weeks prior to sample collection. Patients comprised 117 individuals with SCD, of whom 59 were SS (30 males and 29 females) aged 16 ± 9.9 years (mean ± SD), 25 were AS (14 males and 11 females) aged 21.2 ± 9.1 years (mean ± SD), and 33 were S/β thalassemia (18 males and 15 females) aged 15.1 ± 5.1 years (mean ± SD). The control group included 118 apparently healthy individuals (77 males and 41 females) aged 20.6 ± 5.5 years (mean ± SD). The inclusion criteria for controls were: individuals did not experience any past or current thrombotic events or had a family history of venous or arterial thrombosis (including stroke, deep venous thrombosis or pulmonary embolism), and were recruited either from blood donors, or medical staff. Blood samples were collected after a written informed consent was obtained from each patient or their guardians/parents before entry to the study.

### Main outcome measures

The primary outcome measure was the frequency of FVL and Prothrombin G20210A mutations among SCD compared to controls. Secondary outcomes included type and frequency of clinical complications among SCD positive for FVL or/and Prothrombin G20210A mutations compared to those with wild type genotype.

### Laboratory methods

Hematological data including complete blood counts and red cell indices were measured using a cell counter Sysmex Kx21 (Kobe, Japan) for all study patients and controls.

The sickle cell phenotype was diagnosed by conventional electrophoresis methods (cellulose acetate at alkaline and acid pH) [8]. In addition, hemoglobin electrophoresis was performed for controls to ascertain absence of β-thalassemia trait. DNA was isolated from peripheral blood leukocytes with the QIAamp Blood kit (QIAGEN, Heldin/ Germany) according to manufacturer’s recommendations and kept at −20 °C until analyzed. DNA analysis was used to confirm the diagnosis of sickle cell anemia, sickle cell trait and sickle β-Thalassemia. Homozygosity or heterozygosity for the β^S^ mutation was ascertained by polymerase chain reaction (PCR) followed by digestion with the restriction enzyme DdeI [9]. The β^S^ haplotype was determined by RFLP PCR as described earlier [10]. The Benin β^S^ haplotype was the most predominant haplotype among our patients corresponding to about 88% of all haplotypes. The β-thalassemia alleles in S/βthal patients were screened by PCR reverse dot blot (PCR-RDB) technique [11].

### Detection of factor V Leiden mutation

The FVL G1691A mutation was identified as described earlier [6]. Briefly, a 267-bp fragment of exon 10 of coagulation factor V gene was amplified using the following primer pair: forward primer 5’-TGC CCA GTG CTT AAC AAG ACC A-3′; reverse primer 5′-TGT TAT CAC ACT GGT GCT AA-3′. The PCR product was digested with MnlI restriction enzyme and analyzed on a 3% agarose gel. The normal G allele was confirmed by the presence of three fragments (163, 67, and 37 bp), while the mutant A allele was confirmed by the presence of two fragments (200 and 67 bp).

### Detection of prothrombin G20210A mutation

The prothrombin G20210A mutation was identified as described earlier [7]. Briefly, a 345-bp fragment spanning the 3′ sequence of exon 14 and 5′ sequence of 3′-untranslated region of the prothrombin gene was amplified using the primer pair: forward 5’-TCT AGA AAC AGT TGC CTG GC-3′ and reverse 5′-ATA GCA CTG GGA GCA TTG AAG C-3′. The PCR product was digested with HindIII restriction enzyme and analyzed on a 3% agarose gel. The presence of intact 345 bp fragment on the agarose gel indicates the presence of normal G allele, while the presence of two fragments (322 and 23 bp) indicates the presence of mutant A allele.

### Statistics

Allele frequency was calculated using the gene counting method. The observed genotype frequencies for FVL and prothrombin G20210A mutations among patients and healthy individuals were compared and tested for Hardy–Weinberg equilibrium using the Chi-Square test. The significance of the difference of observed alleles and genotypes between the groups was tested using the Chi-Square analysis after grouping individuals as normal and heterozygous/homozygous carriers of the FVL and prothrombin G20210A mutations. Odds ratio (OR), both unadjusted and age- and sex-adjusted as well as their 95% confidence intervals (CI) were calculated using SPSS logistic regression to estimate the relative risk for the disease. A *P*-value < 0.05 was considered statistically significant. The SPSS software package version 15.0 was used for the statistical analysis.

## Results

### Clinical characteristics

The general and hematological characteristics of study patients are summarized in Table 1. The type of β-thalassemia mutation was determined in 26 out of 33 sickle/β-thalassemia patients. Seven different mutations were determined: IVS-I-1 (G → A), IVS-II-1 (G → A), Codon 39 (C → T), Fs8 (−AA), Codon 30 (AGG → ACG), IVS-I-110 (G → A), and Codon 37 (G → A). Around 80% of the later mutations were associated with β^0^-thalassemia. Relevant clinical information was recorded in all SCD patients. Vascular occlusive cerebral disease (stroke) was diagnosed in four patients based on computerized tomography and focal neurologic defect. One patient out of four was with Sickle/β^0^-thalassemia with IVS-II-1 (G → A) mutation, the remaining three patients were with sickle cell anemia (SS). In addition, three patients were diagnosed with venous thrombosis. The later three patients developed a thrombosis associated with central vein catheter; the diagnosis was based on clinical data. For chronic complications leg ulcers were present in nine patients (6 males, 3 females), all were with sickle cell anemia (SS). X-ray documented avascular bone necrosis (AVN) in seven patients (4 females, 3 males), two of them were with Sickle/β^0^-thalassemia with IVS-II-1 (G → A) and Codon 39 (C → T) mutations. Priapism occurred at the post-pubertal age in two males presenting with sickle cell anemia (SS) (Table 1).

**Table 1:** Charecteristics of SS, AS, S/β^Thal^ patients and controls. Data are presented as mean ± SD for the age and hematological data and frequency for sex and clinical complications

	AS (*n* = 25)	SS (*n* = 59)	S/βThal (*n* = 33)	Controls (*n* = 118)
Sex, F/M	11/14	29/30	15/18	41/77
Age, years	21.2 ± 9.1	16 ± 9.9	15.1 ± 5.1	20.6 ± 5.5
Hematological data				
Hb (mg/dL)	12.9 ± 1.67	8.4 ± 1.09	8.32 ± 1.33	14.1 ± 1.58
MCV (fL)	81.4 ± 7.39	87.4 ± 8.0	69.16 ± 7.91	85.2 ± 4.08
MCH (pg)	26.78 ± 2.91	28.7 ± 2.88	23.34 ± 3.41	31.1 ± 2.76
HbF (%)	1.1 ± 0.63	5.14 ± 3.3	7.98 ± 3.43	0.9 ± 0.23
HbS (%)	36.3 ± 6.1	86.6 ± 7.4	69.8 ± 10.56	0
Hb A2 (%)	2.61 ± 0.41	2.1 ± 0.52	4.46 ± 0.74	2.4 ± 0.52
Clinical complications				
DVT	0	0	3	0
Stroke	0	3	1	0
Priapism	0	2	0	0
Leg ulcers	0	9	0	0
AVN	0	5	2	0

*Hb* hemoglobin, *MCV* mean corpuscular volume, *MCH* mean corpuscular hemoglobin, *HbF* hemoglobin F, *HbS* hemoglobin S, *HbA2* hemoglobin A2. *AS* Sickle cell trait, *SS* Sickle cell anemia, *S/βThal* Sickle β-thalassemia, *DVT* deep vein thrombosis, *AVN* avascular bone necrosis

### Analysis of factor V Leiden G1691A mutation

The frequency of FVL mutation in the study population and its association with SCD is summarized in Table 2. The FVL mutation in its homozygous and heterozygous forms was found in 20 out of 117 patients for an overall prevalence of 17%. Analysis of the SS patients for the FVL mutation revealed that 11 of 59 (18.64%) SS patients were heterozygotes (frequency of A allele 9.32%), while none of the SS patients were homozygote for this mutation. Among the AS patients, one patient was heterozygote and one patient was homozygote for the FVL mutation with a prevalence of 8% and the frequency of A allele was 6%. Analysis of the S/βthal patients revealed that 4 patients were heterozygotes and three were homozygotes for the FVL mutation with a prevalence of 21.21% and the frequency of A allele was 30.3%. Among the control group, 18 individuals were heterozygotes and two were homozygotes for the FVL mutation (16.94%) and the frequency of A allele was 9.32%. Frequency of FVL genotypes in SS, AS and S/βthal patients and controls revealed no statistically significant difference when compared with the predicted genotypes from the Hardy–Weinberg equilibrium (*P* values 0.92, 0.90, 0.89, and 0.72, respectively). Analysis of the association of gender with FVL mutation showed that among the SS patients, four males and seven females were heterozygotes of FVL mutation. While among the controls two males were homozygotes and 18 females were heterozygotes for FVL mutation. In the AS group, one male was heterozygote and another one was homozygote for the FVL mutation. While in S/βthal patients, 2 females and 2 males were heterozygotes and 1 female and 2 males were homozygote for the FVL mutation. Taking the results of FVL mutation altogether, a significantly high prevalence of FVL was observed in Palestinian SS patients compared to controls.

**Table 2:** Prevalence of factor V Leiden mutation and its association with SCD in Palestinian patients

Patient (*n*)	FV Leiden			Crude		Adjusted	
	G/G	G/A	A/A	OR (95% CI)	*P* value	OR (95% CI)	*P* value
SS (59)	48	11	0	9.31(2.1–61.9)	0.027	5.6 (1.91–39.4)	0.039
AS (25)	23	1	1	8.4 (1.12–64.7)	0.038	3.97 (0.51–28.6)	0.17
S/βThal (33)	26	4	3	7.2 (0.63–75.4)	0.19	3.59 (0.35–41.6)	0.26
Controls	98	18	2	1	–	1	–

Logistic regression analysis of age- and sex-adjusted data revealed a significant association between FVL mutation and sickle cell anemia (OR = 5.6; 95% CI = 1.91–39.4, *P* = 0.039) in SS patients (Table 2). However, elevated prevalence of FVL in AS subjects and S/βthal patients was not statistically significant when compared to controls (OR = 3.97, 95% CI = 0.51–28.6, *P* = 0.17 and OR = 3.59, 95% CI = 0.35–41.6, *P* = 0.26, respectively).

Heterozygous FVL mutation was reported in one patient out of seven (14%) with AVN documented by X-ray, and the remaining 6 patients with AVN were normal for this mutation. The later differences were not statistically significant.

### Analysis of the prothrombin G20210A mutation

The frequency of prothrombin G20210A mutation among the study population and its association with SCD is summarized in Table 3. None of the SCD patients were homozygous for prothrombin G20210A mutation, but 8 of the 117 patients were heterozygous for the mutation for an overall prevalence of 6.83%. Heterozygous prothrombin G20210A mutation was found in 3 of 59 (5.08%) SS patients (allele A frequency 2.54%), one of 25 (4%) AS subjects (allele A frequency 2%), 4 of 33 (12.12%) S/βthal patients (allele A frequency 6.06%) and 6 of 118 (5.08%) controls (allele A frequency 2.54%). The frequency of prothrombin G20210A genotypes in SS, AS, and S/βthal patients and controls revealed no significant difference when compared to the predicted genotypes from the Hardy–Weinberg equilibrium (*P* values 0.95, 0.92, 0.91, and 0.83, respectively). In SS patients, 3 males were carriers of prothrombin G20210A mutation while in 118 controls, 2 females possessed this mutation. In AS subjects one male carried the prothrombin G20210A mutation. While in S/βthal patients the mutation was found in 2 females and 2 males.

**Table 3:** Prevalence of prothrombin G20210A mutation and its association with SCD in Palestinian patients

Patient (*n*)	Prothrombin G20210A			Crude		Adjusted	
	G/G	G/A	A/A	OR (95% CI)	*P* value	OR (95% CI)	*P* value
SS (59)	56	3	0	8.4(1.8–56.4)	0.16	6.3(1.17–33.9)	0.12
AS (25)	24	1	0	5.5(1.1–66.2)	0.13	3.71(0.46–26.1)	0.18
S/Thal (33)	29	4	0	9.4(1.9–54.4)	0.09	3.39(0.33–43.4)	0.21
Controls	112	6	0	1	–	1	–

Logistic regression analysis of age- and sex-adjusted data showed that the prevalence of the prothrombin G20210A in SS, AS individuals and S/βthal patients, was not statistically significant compared to controls (OR 6.3, 95% CI 1.17–33.9, *P* = 0.12, OR 3.71, 95% CI 0.46–26.1, *P* = 0.18 and OR 3.39, 95% CI 0.33–43.4, *P* = 0.21, respectively) (Table 3).

Heterozygous prothrombin G20210A mutation was found in one of the 4 patients (25%) with stroke, and the remaining 3 patients with stroke were normal for this mutation. But, the later differences were not statistically significant.

### Clinical symptoms of SCD patients and thrombophilic mutations

The clinical symptoms of SCD patients enrolled in this study are shown in Table 4. The association between clinical manifestation of SCD patients and the co-inheritance of either the FVL or mutant allele of prothrombin G20210A was explored by chi-square analysis (Table 4). SCD patients having the FVL mutation showed a significantly higher incidence of pain in joints, chest and abdomen, as well as regular dependence on blood transfusion compared to SCD patients with the wild-type genotype. However, SCD patients having the mutant allele for prothrombin G20210A mutation showed no significant association with the clinical manifestations investigated in this study, except for blood transfusion, when compared to SCD patients with the wild-type genotype.

**Table 4:** Clinical symptoms observed among SCD patients with the FVL G1691A and prothrombin G20210A mutations versus SCD patients without these mutations. Results are expressed as frequency and percentages

	FVL mutant (*N* = 20)	FVL wild (*N* = 97)	*P*-value	Prothrombin mutant (*N* = 8)	Prothrombin wild (*N* = 109)	*P* value
Joints pain	16 (80%)	41 (42.2%)	0.0132	4 (50%)	55 (50.4%)	NS^a^
Chest pain	18 (90%)	52 (53.6%)	0.006	4 (50%)	57 (52.2%)	NS
Abdominal pain	14 (70%)	48 (49.5%)	0.0311	4 (50%)	61 (55.9%)	NS
Splenomegaly	18 (90%)	53 (54.6%)	0.0071	3 (37.5%)	49 (44.9%)	NS
Frequency of Blood transfusion (no./year)						
0–2	2 (10%)	21 (21.6%)	NS	1 (12.5%)	29 (26.6%)	NS
3–5	3 (15%)	7 (7.2%)	0.047	2 (25%)	45 (41.3%)	NS
6–9	11 (55%)	4 (4.1%)	0.018	4 (50%)	23 (21.1%)	0.003
≥ 10	1 (5%)	0 (0%)	NS	-------	-------	-------

^a^*NS* not significant

## Discussion

Thrombosis is a common complication in patients with sickle cell disease, where coagulation and fibrinolytic abnormalities lead to the development of a hypercoagulability state in such patients [12]. Acute chest syndrome and occlusive strokes have been evidenced in patients with SCD as major causes of death, which results from previous thromboembolism events [3].

FVL and prothrombin G20210A are the major inherited risk factors for venous thrombosis and their presence increases the risk of thrombosis 5–10-folds among patients with deep venous thrombosis [13].

Nowadays, there is no single clinical or laboratory test that can predict which patients are at high risk for developing thrombotic complications of SCD. Inherited predisposition factors to thrombosis could coexist with other endothelial, erythrocyte, and coagulation abnormalities and leads to an increased risk of thrombotic complications [14]. However, the role of inherited thrombophilia in the pathogenesis of SCD has been tackled by few studies but the findings were not conclusive or even controversial. For example, a low prevalence of FVL and prothrombin G20210A was reported in SCD patients from Saudi Arabia representing Saudi nationals [15], Brazilians with African descent [16] or African Americans in the United States [17]. In contrast, a high prevalence of FVL but not prothrombin G20210A was reported among Indian [18] and Iranian SCD patients [19]. In the present study, a significantly higher prevalence of FVL was observed among SS patients compared to controls while the frequency of prothrombin G20210A mutation was not significantly different among SCD patients compared to controls. Our findings were consistent with previous reports concerning FVL and prothrombin G20210A [18, 19] but in contrast to other studies [15–17]. The different findings concerning prevalence of FVL and prothrombin G20210A among SCD patients may be partially due to the different genetic background of the different ethnicities of study patients as well as the limited sample size in some studies.

In the present study, we also examined the role of inherited mutations FVL and prothrombin G20210A in the thrombotic complications of Palestinian SCD patients. Inherited risk factors for vascular disease, including venous as well as arterial thrombosis, have been described in the world population [20, 21]. However, the risk factor resulting from the prothrombin gene variant was distributed similarly among patients of Caucasian descent as well as patients of African descent [22].

In this study, the development of thrombosis and occlusive stroke were compared and no distinct prevalence of the risk factors was found. This is in agreement with the lack of correlation between FVL mutation and cerebral ischemia among patients with SCD [17], as well as in the general population. Also, the presence of chronic complications was not related to the presence of risk factors studied. In addition, our results indicated a higher incidence of pain and increased dependence on blood transfusion among SCD patients with FVL. While SCD patients with prothrombin G20210A mutation showed a significant association with increased dependence on blood transfusion but no significant association with pain in chest and joints and splenomegaly.

The high prevalence rate of FVL among healthy subjects from Palestine was interestingly matched with similarly high rates from neighboring Jordan [23], Israeli Arabs [24] and Lebanon [25]. This suggested that FVL mutation must have originated as a single mutational event outside of Europe, then spreading by migration of mutation-carrying individuals [26].

Prothrombin G20210A mutation was also present among healthy controls from Palestine, but at a lower frequency than FVL. Our results (2.54%) were comparable to that reported for communities of Caucasian descent, including Turkey (2.7%) [27] and Italy (3.2%) [28].

## Conclusions

This study is the first report that shows the prevalence and clinical impact of FVL and prothrombin G20210A mutations among Palestinian SCD patients. FVL was more prevalent among SS patients compared to normal subjects (control group). SCD patients with FVL showed a significantly higher incidence of pain in chest, abdomen and bone joints making these SCD patients dependent on regular blood transfusion to modify the vasoocclusive crises. The high frequency of FVL and its significant correlation with sickle cell anemia from Palestine could be an important risk factor for developing occlusive crisis. Studies that include a larger number of patients and controls are necessary to define specific guidelines. It is still possible that other inherited thrombophilic mutations may contribute to thrombotic complications in SCD. Mutations and polymorphisms in the fibrinogen gene, C677T mutation in the methylenetetrahydrofolate reductase (MTHFR) gene, C1565T mutation in the platelet glycoprotein IIIa (GPIIIa) gene, and factor VII gene and others should be analyzed to determine the contribution of inherited thrombophilic mutations to thrombotic complications in patients with SCD.

## Abbreviations

AS: Sickle cell trait
AVN: Avascular bone necrosis
CI: Confidence interval
FVL: Factor V Leiden
GPIIIa: Platelet glycoprotein IIIa gene
MTHFR: Methylene tetrahydrofolate reductase
OR: Odds ratio
RDB-PCR: Reverse dot blot polymerase chain reaction
RFLP PCR: Restriction fragment length polymorphism – polymerase chain reaction
SCD: Sickle cell disease
SS: Homozygote sickle cell anemia
β^S^: Hemoglobin S
